# Dietary intake patterns of children aged 6 years and their association with socioeconomic and demographic characteristics, early feeding practices and body mass index

**DOI:** 10.1186/s12889-016-3725-2

**Published:** 2016-10-06

**Authors:** Leonardo Pozza Santos, Maria Cecília Formoso Assunção, Alicia Matijasevich, Iná S. Santos, Aluísio J. D. Barros

**Affiliations:** 1 Postgraduate Program in Epidemiology, Federal University of Pelotas, 1160 Marechal Deodoro Street, 3th floor, Pelotas, Zip Code 96020-220 Brazil; 2 Department of Preventive Medicine, University of São Paulo, 455 Dr Arnaldo avenue, 2nd floor, São Paulo, Zip Code 01246-903 Brazil

**Keywords:** Dietary intake, Principal component analysis, Cohort studies, Nutrition assessment

## Abstract

**Background:**

Dietary intake patterns of children from the 2004 Pelotas birth cohort study have been described at 12, 24 and 48 months of age, but there is no information about dietary patterns of these children at 6 years. Then, we aimed to identify and describe dietary intake patterns of children aged 6 years as well as to assess their association with socioeconomic and demographic characteristics, early feeding practices and BMI z-score at 6 years.

**Methods:**

We used principal components analysis to identify dietary intake patterns of 3,427 children from the 2004 Pelotas (Brazil) birth cohort study. We used multiple linear regression models to evaluate whether socioeconomic and demographic characteristics (socioeconomic position, mother’s age at birth, and child’s sex and skin colour), early feeding practices (exclusive breastfeeding duration and age of introduction of complementary foods), and BMI z-score at 6 years were associated with dietary intake patterns.

**Results:**

We identified seven dietary components of children’s dietary intake patterns, namely: *fruits and vegetables*, *snacks and treats*, *coffee and bread*, *milk*, *cheese and processed meats*, *rice and beans* and *carbohydrates*. Dietary patterns were socially patterned, since six dietary components were associated with socioeconomic position. Moreover, high intake of *snacks and treats* and less *fruits and vegetables* were associated with children born to teenage mothers, with those exclusively breastfed for less than one month, and with those who started on complementary feeding before 4 months. Finally, overweight and obese children at 6 years presented lower intake of four out of seven dietary components, but we need to be cautious in interpretation due to limitations on food consumption reporting and due to possible reverse causality.

**Conclusion:**

Dietary intake patterns in children are strongly influenced by socioeconomic characteristics. Other factors such as younger maternal age at birth, and both early weaning and early introduction of complementary feeding appear to be related with ‘unhealthier’ patterns. Overweight and obese children presented lower intake of four out of seven dietary components, but further studies would be interesting to understand the longitudinal effect of children’s feeding practices on BMI and adiposity.

**Electronic supplementary material:**

The online version of this article (doi:10.1186/s12889-016-3725-2) contains supplementary material, which is available to authorized users.

## Background

Overweight and obesity are a concerning issue in childhood, as more than 40 million children around the world face this problem, of whom 80 % are from low- and middle-income countries [1, 2]. Due to the high rates of childhood obesity worldwide, it is extremely important to study and understand the dietary intake patterns in childhood, as early feeding practices can play a major role in the development of obesity and chronic diseases in the short and long term [3–5].

Principal component analysis (PCA) is *a posteriori* method to measure dietary intake patterns in epidemiological studies, and has emerged as a complementary approach to study feeding practices in children and adults. In summary, PCA reduces a large amount of food data, examining inter-correlations among foods or nutrients, and allows investigators to assess dietary intake patterns and their associations with risk factors and health conditions [6–8].

PCA has been used to describe feeding practices in children, adolescents and adults as well as to investigate their relationships and their role as risk factors for chronic diseases [9–13]. Previous investigations often reported strong association between dietary intake patterns and socioeconomic and demographic characteristics in childhood [9, 10, 14, 15], while results for the association between dietary intake patterns and obesity status are inconsistent [16–18].

The dietary intake patterns of children from the 2004 Pelotas birth cohort study have already been described by PCA at ages 12, 24 and 48 months [19]. However, there is no published information about dietary intake patterns at 6 years, when another follow-up of this cohort was conducted. Unlike younger children, 6 year-old children are more capable of choosing their own foods, and factors such as school environment and greater interaction with other children can also affect their food choices [20].

In order to contribute to the body of knowledge about dietary intake patterns in early childhood, we aimed to identify and describe the dietary intake patterns of 6 year-old children from the 2004 Pelotas birth cohort study and to assess their association with socioeconomic and demographic characteristics, early feeding practices and body mass index (BMI).

## Methods

### Study participants

Pelotas is a municipality situated in Southern Brazil with 330,000 inhabitants, according to the last Brazilian Demographic Census. Compared to Brazil as a whole, Pelotas presents higher Human Development Index (0.739 vs. 0.699), but lower Gross Domestic Product per capita (17,353.13 *reais* (Brazil’s currency) vs. 26,445.00 *reais*), and lower illiteracy (4.1 % vs. 8.5 %).

In 2004, a birth cohort study was started in Pelotas, and 4,231 newborns were recruited, accounting for 99.2 % of all births to mothers living in the urban area of the city. All newborns were assessed and their mothers were interviewed within 24 h after birth. A structured questionnaire comprising nine sections was administered by trained interviewers and information about the child, mother and family, current pregnancy, and birth was collected. The cohort children were then followed up at the age of 3, 12, 24 and 48 months, and 6 years. In all follow-ups, information about anthropometric variables (weight, length and/or height), health and nutritional status, child development, housing conditions and socioeconomic position (SEP) were collected. The follow-up rates at 3, 12, 24 and 48 months, and 6 years were 95.7, 94.2, 93.4, 91.8 and 90.2 %, respectively. Details of the methods used in the perinatal studies and subsequent follow-ups were reported previously [21, 22].

### Food consumption at 6 years

Between 2010 and 2011 children from the 2004 Pelotas birth cohort study were followed-up when the average age of the sample was 6.8 years. In this follow-up, food consumption was assessed using a Food Frequency Questionnaire (FFQ) administered to the children’s mothers. The FFQ consisted of 54 food items divided into 9 food groups (cereals, pulses, vegetables, fruits, milk and dairy products, meat and meat products, fats, sugars and others) for a 12-month recall period. It was a semi-quantitative FFQ with open-ended questions and nine frequency categories. If the answer was “yes”, the mothers were then asked the children’s food consumption frequency (per day, month or year) as well as the portion size consumed (small, medium, large or extra-large) in relation to a medium portion size of determined food, as indicated in the FFQ. A medium portion size was the median food consumption based on a 24-h dietary recall applied in a group of children with similar age of our children [23]. A small portion was half a medium portion; a large portion was two times a medium portion; and an extra-large portion was two and a half times a medium portion. The FFQ used in our study was validated in a sample of children aged 1 to 6 years from Pelotas, and based on three 24-h dietary recalls. In the validation study, Pearson correlation was 0.50 or more for macronutrients, calcium, iron, sodium, vitamin C, cholesterol and saturated fat (unpublished results).

The 54 food items included in the FFQ were categorized into 22 food groups based on their nutritional similarities (Additional file 2: Table S1) to aid the understanding and interpretation of results. The main rationale was to group similar foods consumed in the same way. For instance, group 7 included vegetables mainly consumed raw in salads, and group 8 included vegetables that are mainly cooked. Chocolate milk powder was categorized separately from candies and chocolate bars because it is offered to children mixed with milk as part of a meal rather than on its own as a treat.

### Socioeconomic and demographic characteristics, early feeding practices and BMI at 6 years

In this study, we used information about socioeconomic and demographic characteristics as well as early feeding practices and children’s BMI. Socioeconomic information was based on quintiles of SEP, according to the Brazilian National Economic Index (IEN) [24]. IEN is an index which calculates wealth scores based on assets and household head education according to Brazilian Demographic Census data. Children’s demographic characteristics were based on maternal age at birth (18–35 years, less than 18 years and more than 35 years), child’s sex (male/female), and parent reported skin colour (white, brown and black).

Early feeding practices were characterized by exclusive breastfeeding duration and age of introduction of complementary foods. As just 5 % of children were never exclusively breastfed, we categorized this variable as following: 0–7 days, 8 days - <1 month, 1 - <3 months and ≥3 months. Age of introduction of complementary foods was categorized as ≥4 months, 1–3.9 months and less than 1 month.

The latest follow-up of the 2004 birth cohort study included measures of weight and height. Weight was measured by a high precision scale (0.01 kg) that was part of the BODPOD machine (Cosmed, Italy, http://goo.gl/7jzfLc). Height was measured twice by trained anthropometrists using a Harpenden metal stadiometer, with 1 mm precision (Holtain, Crymych, UK). We then calculated BMI by dividing weight (kg) by height (m^2^), and standardized it according to the World Health Organisation (WHO) 2007 reference [25]. Finally children were classified according to BMI z-score (‘normal weight’ -2 to ≤ +1 SD/‘overweight’ > +1 to ≤ +2 SD/‘obese’ > +2 SD).

### Statistical analyses

To make our results comparable with previous dietary intake patterns of our cohort [19], we have chosen PCA as statistical method to be used in our analyses. PCA was performed using 22 variables corresponding to the food groups described above. These food groups included in PCA were based on the initial 54 food items included in FFQ (Additional file 2: Table S1), and were expressed in consumption frequency per year. The number of components selected from PCA was based on screeplot and on the components with eigenvalues greater than 1. We used a rule-free approach to PCA and considered representative of each component all food items with a factor loading greater than 0.3. A varimax rotation was then applied to obtain components with a near zero or maximum loading to improve component interpretability. Component scores were calculated for each child in the sample and standardized (mean = 0 and s.d. = 1), providing a more appropriate scale. The labelling of dietary intake components was based on characteristics and nutritional aspects of the food groups selected in each component.

We conducted multiple linear regression models to assess whether SEP, maternal age at birth, children’s sex and skin colour, early feeding practices, and BMI status at 6 years were associated with dietary intake patterns scores. As these explanatory variables are in different levels in the pathway of association with dietary intake patterns (Additional file 3: Figure S1), we conducted multiple linear regression models in 4 different steps, in order to control adequately for confounding. In addition, all the models were also adjusted for both sex and total of kilocalories consumed per day, since daily energy intake presented strong association with all components identified.

The four steps of our linear regression models are described below:

Step 1) When the independent variable was SEP the model was adjusted for sex and daily energy intake;
Step 2) When the independent variables were demographic characteristics (mother’s age at birth, children’s sex and skin colour) the model was adjusted for SEP, sex (except when sex was the independent variable) and daily energy intake;
Step 3) When the independent variables were early feeding practices (exclusive breastfeeding and age of introduction of complementary feeding) the model was adjusted for SEP, mother’s age at birth, child’s sex and skin colour, and daily energy intake; and
Step 4) When the independent variable was BMI status, the model was adjusted for SEP, mother’s age at birth, child’s sex and skin colour, exclusive breastfeeding duration, and daily energy intake.

As in the majority of investigations studying the relationship between dietary intake patterns and obesity status BMI is usually treated as the outcome, we conducted further analyses treating BMI as outcome and dietary intake components as exposure, in order to see if the dietary intake components identified in our study are associated with lower or higher BMI z-score at 6 years. We categorised dietary components scores in tertiles, representing low (1^st^ tertile), intermediate (2^nd^ tertile) or high (3^rd^ tertile) consumption, and performed crude and adjusted linear regression. The confounders included in this analysis were the same included in *Step 4* of our linear regression model (SEP, mother’s age at birth, child’s sex and skin colour, exclusive breastfeeding duration, and daily energy intake).

It is important to highlight that we only considered a possible confounder in the different steps above those variables associated with both exposure and outcomes in the crude model (*p*-value <0.20). We evaluated multicollinearity in the model using the variance inflation factor. Due to high correlation between exclusive breastfeeding duration and age of introduction of complementary feeding, we included only exclusive breastfeeding duration in step 4 in order to avoid multicollinearity.

All the analyses were performed using Stata version 13.1 (Stata Corp., College Station, TX, USA).

## Results

We included 3,427 children in the analysis for whom FFQ information was available. Children followed up at 6 years differed from those lost to follow up in terms of SEP, exclusive breastfeeding, and age of introduction of complementary foods. Children lost were poorer, exclusively breastfed for less time, and started on complementary feeding earlier than those who participated in the last follow-up.

Table 1 presents the characteristics of children included in the study. Regarding SEP, 23 % of children were in the first (poorest) quintile according to the Brazilian IEN, while 17 % were classified in the last (richest) quintile. In addition, almost 10 % of the children were born to teenage mothers and 10.7 % were born to mothers who were older than 35 years at the time of birth. More than 70 % of children were white according to mother’s report and only 21 % were exclusively breastfed for 3 months or more. Regarding age of introduction of complementary foods, 1/3 of the children started on complementary feeding before 1 month, and approximately 35 % of the children were classified as overweight or obese at 6 years according to the WHO 2007 reference (Table 1). Finally, the mean of daily energy intake was 1,594.7 Kcal, and boys presented higher daily energy intake compared to girls (1,625.5 Kcal vs. 1,561.9 Kcal) (data not shown).

**Table 1:** Sample characteristics according to independent variables (Number of participants and percentage). Pelotas 2004 birth cohort study

Variables	*N*	%
SEP (quintiles)		
1^st^ (lower)	784	22.9
2^nd^	716	21.0
3^rd^	766	22.4
4^th^	553	16.2
5^th^ (higher)	595	17.4
Maternal age at birth (years)		
18–35	2733	79.8
< 18	324	9.5
> 35	367	10.7
Children’s sex		
Male	1772	51.9
Female	1645	48.1
Children’s skin colour		
White	2324	71.4
Brown	502	15.4
Black	428	13.2
Exclusive breastfeeding		
0–7 days	859	25.4
8 days - <1 month	375	11.1
1 - <3 months	1421	42.1
≥ 3 months	721	21.4
Age of introduction of complementary food		
≥ 4 months	498	14.8
1–3.9 months	1685	49.9
< 1 month	1190	35.3
BMI at 6 years (z-score)		
Normal	2016	65.0
Overweight	555	17.9
Obese	533	17.2

*SEP* Socioeconomic position, *BMI* Body mass index

Although all food items were used to calculate dietary component scores, our results show only the representative food items in each component (Table 2). We identified seven dietary components of children’s dietary intake patterns at 6 years which explained almost 50 % of the total variance in food consumption. The first component, which we called *fruits and vegetables*, included raw vegetables (lettuce, tomato, carrot, beet and chayote), cooked vegetables (cabbage, broccoli, carrot, beet, and chayote), fruits and fresh fruit juice. The second component, *snacks and treats*, included candies, sweetened beverages and crisps (Table 2).

**Table 2:**
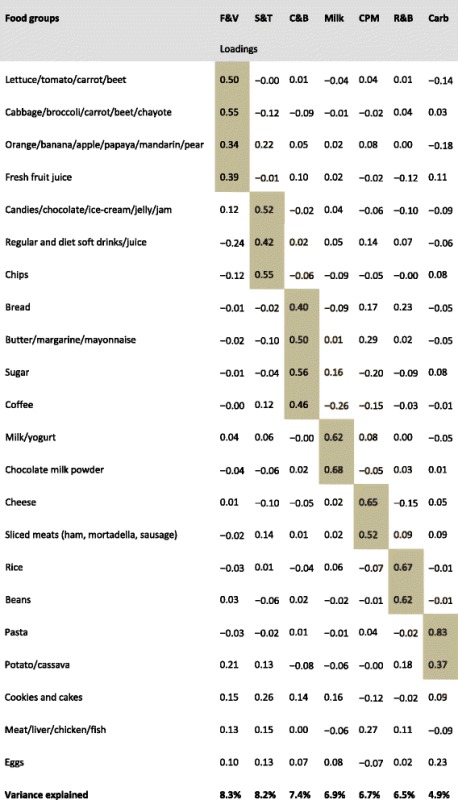
Children’s dietary intake components and their factor loadings at 6 years. The 2004 Pelotas birth cohort study

*F & V* fruits and vegetables, *S & T* snacks and treats, *C & B* coffee and bread, *CPM* Cheese and processed meats, *R & B* rice and beans, *Carb* Carbohydrates

In several countries, coffee consumption is uncommon in young populations, but this is not the case in Brazil [26]. From early on, Brazilian children have the habit of drinking coffee (with sugar added) and having bread and margarine or butter for breakfast or afternoon snack. Given this practice, we named the third component *coffee and bread*. This component included bread, butter, mayonnaise, margarine, coffee and sugar (Table 2).

The fourth component, *milk*, included milk drinks and chocolate milk powder. The fifth component, *cheese and processed meats*, included cheese, sliced meats (e.g. ham) and sausages. The sixth component, *rice and beans*, included rice and beans, which represent a ubiquitous combination in the Brazilian diet, consumed by all population strata at lunch and/or dinner, and frequently served with some meat and salads. Lastly, the seventh component, *carbohydrates*, included pasta, potato and cassava. All food items included in the seven dietary components showed positive loadings (Table 2).

Results on the association between dietary patterns and SEP, adjusted for total daily energy intake and sex are shown in Table 3. SEP was strongly associated with six out of seven dietary components. The higher SEP the lower intake of *fruits and vegetables*, *snacks and treats*, *coffee and bread* and *rice and beans*. In contrast, SEP was positively associated with consumption of *milk* and *cheese and processed meats*. There was no difference in consumption of *carbohydrates* according to SEP.

**Table 3:** Multiple linear regression between dietary intake components at 6 years, and socioeconomic and demographic characteristics, and early feeding practices. Pelotas 2004 birth cohort study (*N* = 3,427)

Variable	Fruits and Vegetables	*p*-value	Snack and treats	*p*-value	Coffee and Bread	*p*-value	Milk	*p*-value
	β (CI 95 %)		β (CI 95 %)		β (CI 95 %)		β (CI 95 %)	
SEP quintiles^a^		0.083		<0.001		<0.001		<0.001
1^st^ (lower)	0.00		0.00		0.00		0.00	
2^nd^	−0.07 (−0.17; 0.03)		−0.09 (−0.17; −0.01)		−0.17 (−0.27; −0.07)		0.22 (0.12; 0.32)	
3^rd^	−0.06 (−0.16; 0.04)		−0.14 (−0.21; −0.06)		−0.26 (−0.35; −0.17)		0.40 (0.30; 0.50)	
4^th^	−0.04 (−0.14; 0.07)		−0.13 (−0.22; −0.05)		−0.43 (−0.52; −0.33)		0.45 (0.35; 0.56)	
5^th^ (upper)	−0.11 (−0.20; −0.01)		−0.26 (−0.33; −0.19)		−0.51 (−0.60; −0.42)		0.46 (0.36; 0.57)	
Maternal age at birth^b^		0.211		0.404		0.209		0.406
18-35 years	0.00		0.00		0.00		0.00	
< 18 years	−0.13 (−0.23; −0.02)		0.14 (0.04; 0.24)		−0.07 (−0.18; 0.04)		−0.03 (−0.14; 0.08)	
> 35 years	0.12 (0.02; 0.21)		−0.01 (−0.10; 0.07)		−0.03 (−0.11; 0.05)		−0.03 (−0.13; 0.06)	
Sex^b^		0.037		0.383		0.113		<0.001
Male	0.00		0.00		0.00		0.00	
Female	0.07 (0.01; 0.13)		0.02 (−0.03; 0.07)		−0.05 (−0.11; 0.01)		−0.13 (−0.20; −0.07)	
Skin color^b^		0.627		0.080		0.017		0.041
White	0.00		0.00		0.00		0.00	
Brown	0.00 (−0.09; 0.09)		0.06 (−0.01; 0.14)		0.08 (−0.01; 0.17)		0.06 (−0.15; 0.04)	
Black	0.10 (−0.01; 0.20)		0.04 (−0.04; 0.12)		0.16 (0.07; 0.26)		−0.19 (−0.29; −0.09)	
Exclusive breastfeeding duration^c^		0.004		0.004		0.034		0.419
≤ 7 days	0.00		0.00		0.00		0.00	
8 days - <1 month	−0.04 (−0.14; 0.07)		−0.07 (−0.15; 0.01)		−0.01 (−0.10; 0.12)		0.05 (−0.07; 0.17)	
1 month - <3 months	0.02 (−0.06; 0.10)		−0.04 (−0.11; 0.02)		0.01 (−0.07; 0.09)		−0.01 (−0.09; 0.09)	
≥ 3 months	0.13 (0.04; 0.21)		−0.11 (−0.18; −0.04)		−0.10 (−0.18; −0.02)		0.05 (−0.04; 0.14)	
Age of introduction of complementary foods^c^		0.001		0.031		0.022		0.372
≥ 4 months	0.00		0.00		0.00		0.00	
1–3.9 months	−0.11 (−0.21; −0.02)		0.04 (−0.02; 0.11)		0.11 (0.03; 0.19)		−0.04 (−0.13; 0.05)	
< 1 month	−0.17 (−0.27; −0.07)		0.08 (0.01; 0.15)		0.12 (0.04; 0.21)		−0.05 (−0.15; 0.05)	
Variable	Cheese and processed meats	*p*-value	Rice and beans	*p*-value	Carbohydrates	*p*-value		
	β (CI 95 %)		β (CI 95 %)		β (CI 95 %)			
SEP quintiles^a^		<0.001		<0.001		0.099		
1^st^ (lower)	0.00		0.00		0.00			
2^nd^	0.17 (0.08; 0.26)		−0.19 (−0.29; −0.10)		−0.04 (−0.15; 0.07)			
3^rd^	0.22 (0.13; 0.32)		−0.26 (−0.36; −0.16)		0.01 (−0.10; 0.12)			
4^th^	0.34 (0.23; 0.45)		−0.40 (−0.51; −0.30)		−0.01 (−0.10; 0.10)			
5^th^ (upper)	0.45 (0.36; 0.54)		−0.63 (−0.73; −0.54)		0.08 (−0.02; 0.18)			
Maternal age at birth^b^		0.031		0.020		0.331		
18-35 years	0.00		0.00		0.00			
< 18 years	−0.02 (−0.13; 0.10)		−0.01 (−0.12; 0.09)		−0.03 (−0.13; 0.07)			
> 35 years	−0.10 (−0.19; −0.02)		−0.13 (−0.23; −0.03)		−0.05 (−0.17; 0.07)			
Sex^b^		0.942		0.001		0.058		
Male	0.00		0.00		0.00			
Female	0.00 (−0.07; 0.06)		−0.10 (−0.16; −0.04)		−0.06 (−0.13; 0.00)			
Skin color^b^		0.396		0.002		0.689		
White	0.00		0.00		0.00			
Brown	−0.03 (−0.12; 0.05)		0.14 (0.04; 0.24)		0.04 (−0.07; 0.15)			
Black	−0.03 (−0.12; 0.07)		0.12 (0.02; 0.22)		−0.06 (−0.17; 0.04)			
Exclusive breastfeeding duration^c^		0.061		0.548		0.851		
≤ 7 days	0.00		0.00		0.00			
8 days - <1 month	0.09 (−0.02; 0.21)		0.00 (−0.11; 0.11)		−0.09 (−0.19; 0.01)			
1 month - <3 months	0.03 (−0.05; 0.11)		0.02 (−0.06; 0.11)		0.00 (−0.09; 0.09)			
≥ 3 months	0.10 (0.01; 0.18)		−0.04 (−0.12; 0.05)		−0.01 (−0.10; 0.08)			
Age of introduction of complementary foods^c^		0.092		0.506		0.578		
≥ 4 months	0.00		0.00		0.00			
1–4 months	−0.02 (−0.11; 0.07)		0.07 (−0.01; 0.16)		0.01 (−0.08; 0.10)			
< 1 month	−0.07 (−0.16; 0.02)		0.05 (−0.04; 0.14)		−0.02 (−0.11; 0.07)			

^a^Step 1: adjusted for sex and daily energy intake (kilocalories per day); ^b^Step 2: adjusted for SEP, sex (except when sex was the independent variable) and daily energy intake (kilocalories per day); ^c^Step 3: adjusted for SEP, mother’s age at birth, child’s sex and skin colour, and daily energy intake (kilocalories per day)

*P*-values from trend tests are displayed

*SEP* Socioeconomic position

We also observed that children born to teenage mothers presented lower intake of *fruits and vegetables* (β = −0.13; CI 95 % -0.23;-0.02) and higher intake of *snacks and treats* (β = 0.14; CI 95 % 0.04; 0.24) when compared to children born to adult mothers. On the other hand, children from mothers who were more than 35 years at birth consumed more *fruits and vegetables* (β = 0.12, CI 95 % 0.02;0.21), but less *cheese and processed meats* (β = −0.10; CI 95 % -0.19;-0.02) and *rice and beans* (β = −0.13; CI 95 % -0.23;-0.03) than children born to younger mothers, independently of SEP, daily energy intake and sex (Table 3).

Girls presented lower intake of *coffee and bread* (β = −0.05; CI 95 % -0.11;-0.01), *milk* (β = −0.13; CI 95 % -0.20;-0.07) and *rice and beans* (β = −0.10; CI 95 % -0.10;-0.04), but higher intake of *fruits and vegetables* (β = 0.07; CI 95 % 0.01;0.13) than boys. In addition, black children showed lower intake of *milk* (β = −0.19; CI 95 % -0.29;-0.09) and higher intake of *coffee and bread* (β = 0.16; CI 95 % 0.07;0.26), while brown and black children presented higher intake of *rice and beans* than white children. There was no difference in consumption of *fruits and vegetables*, *snacks and treats*, *cheese and processed meats* and *carbohydrates* according to skin colour (Table 3).

Regarding to children’s early feeding practices, children who were exclusively breastfed for, at least, three months had lower consumption of *snacks and treats* (β = −0.11; CI 95 % -0.18;-0.04) and *coffee and bread* (β = −0.10; CI 95 % -0.18;-0.02), but higher *fruits and vegetables* (β = 0.13; CI 95 % 0.04;0.21) and *cheese and processed meats* (β = 0.10; CI95 0.01;0.18). Children who started on complementary feeding before 4 months of age consumed more *snacks and treats* and *coffee and bread*, but less *fruits and vegetables* at 6 years (Table 3).

Finally, obese children at 6 years presented lower intake of *snacks and treats* (β = −0.07, CI 95 % -0.14;-0.01) compared to non-obese children. In addition, overweight and obese children consumed less *coffee and bread* and *carbohydrates*, and more *cheese and processed meats* when compared to normal BMI children (Table 4).

**Table 4:** Multiple linear regression between BMI status and dietary intake components at 6 years. Pelotas 2004 birth cohort study (*N* = 3,427)

Variable	Fruits and Vegetables	*p*-value	Snack and treats	*p*-value	Coffee and Bread	*p*-value	Milk	*p*-value
	β (CI 95 %)		β (CI 95 %)		β (CI 95 %)		β (CI 95 %)	
BMI z-score^a^		0.040		0.023		0.005		0.843
Normal	0.00		0.00		0.00		0.00	
Overweight	−0.06 (−0.14; 0.03)		−0.03 (−0.09; 0.04)		−0.10 (−0.18; −0.02)		0.05 (−0.04; 0.15)	
Obese	−0.07 (−0.18; 0.00)		−0.07 (−0.14; −0.01)		−0.11 (−0.19; −0.02)		−0.01 (−0.09; 0.08)	
	Cheese and processed meats	*p*-value	Rice and beans	*p*-value	Carbohydrates	*p*-value		
	β (CI 95 %)		β (CI 95 %)		β (CI 95 %)			
BMI z-score^a^		0.001		0.281		0.002		
Normal	0.00		0.00		0.00			
Overweight	0.16 (0.07; 0.25)		0.05 (−0.04; 0.14)		−0.16 (−0.25; −0.08)			
Obese	0.13 (0.04; 0.23)		−0.07 (−0.16; 0.02)		−0.11 (−0.20; −0.02)			

^a^Step 4: Adjusted for SEP, mother’s age at birth, child’s sex and skin colour, exclusive breastfeeding duration, and daily energy intake (kilocalories per day)

*P*-values from trend tests are displayed

*BMI* Body mass index

In extra analysis treating BMI as outcome and dietary intake components as exposure, we observed that high consumption of *coffee and bread* were associated with lower BMI z-score at 6 years, while moderate and high consumption of *cheese and processed meats* increased the mean of BMI z-score at 6 years, independently of socioeconomic and demographic characteristics, exclusive breastfeeding, and daily energy intake (Additional file 1: Table S2).

## Discussion

### Socioeconomic and demographic characteristics

The most notable finding of our study was that the dietary intake patterns varied mostly according to social characteristics, since the bigger coefficients are for SEP. Although we had found associations between the other explanatory variables and some specific dietary intake components, the magnitude of effect for these variables were not as big as seen for SEP.

Higher SEP was associated with lower intake of *snacks and treats* (such as crisps, soft drinks and chocolates), but also with less *fruits and vegetables*. Richer children had more *milk* and less *rice and beans* (the traditional daily food in Brazil), and they also consumed more *cheese and processed meats*. Based on this picture, it was not possible to identify a clear health-oriented pattern among richer children, although the trend of poorer children eating more treats was already seen at earlier ages in this cohort [19].

It seems that the nutritional transition, which started a few decades ago in Brazil, is not yet finished in children from Pelotas, since we could not observe a clear picture associating high SEP with a ‘healthier’ pattern, as seen in other studies [27–29]. Studies with children from high-income countries found a clear indication of a ‘healthy’ dietary intake pattern in richer children, with, for example, higher intake of fruits and vegetables [27, 28], while children from lower SEP present high intake of ‘processed’ pattern [28]. Moreover, a recent review including studies with adults from low- and middle-income countries - among them 9 studies from Brazil - concluded that a ‘healthier’ dietary pattern was associated with higher SEP [29]. Lower intake of *fruits and vegetables* and *snacks and treats* at same time among richer children may be an indication that children from Pelotas are in the midway of a nutritional transition, going from a dietary intake pattern typical of low-income countries to one typical of high-income countries [30].

Maternal age at birth was also associated with dietary intake patterns at 6 years. Children who were born to teenage mothers consumed more *snacks and treats*, characterized by a high intake of candies, soft drinks and crisps, and less *fruits and vegetables*, suggesting unhealthy food consumption. High intake of treats was already associated with younger mothers at 48 months in this cohort [19]. In agreement with our results, two studies conducted with children from the Avon Longitudinal Study of Parents and Children (ALSPAC) found that children from younger mothers (≤20 years) consumed more biscuits, sweets and crisps at 6 and 15 months [10] and more ‘junk’ foods at 3 years [31].

We also observed that children from mothers who were more than 35 years at the time of giving birth consumed more *fruits and vegetables* and less *cheese and processed meats*. Previous studies have reported that children from older mothers presented higher intake of healthy (fruits, vegetables and fish) and traditional (meat and vegetables) components [32, 33].

Here, it is clear that higher maternal age at birth is associated with a healthier dietary intake pattern. As young mothers, especially the teenagers, are less educated and do not have full access to health information, it is important to focus on nutritional counselling to this group in health practice, in order to promote healthier diets and lifestyles among these mothers and their children.

Consistent with other studies from different settings [34–36], boys presented higher intake of *milk* than girls as well as more *coffee and bread*, more *rice and beans*, and more daily energy intake. In contrast, girls presented higher consumption of *fruits and vegetables* than boys. These differences seen in adherence to dietary components may be explained by differences in food preferences according to sex [37]. Furthermore, unpublished results of this cohort showed that boys have higher physical activity than girls at 6 years (measured by accelerometer), which could increase dietary needs in boys, increasing their daily energy intake as well as their adherence to the majority of dietary components. Nevertheless, this is speculative since we did not run any analysis to assess the effect of physical activity on dietary intake in this cohort.

Skin colour was also seen as an important factor associated with dietary intake patterns at 6 years. We observed that non-white children consumed more *coffee and bread* and *rice and beans*, and less *milk*, irrespective of SEP, sex and daily energy intake. Skin colour is an important marker of wealth inequality in Brazil, and the non-white population (mainly the blacks) always present worse results for socioeconomic indicators. This scenario influences food availability at the household level and the number of meals per day, which could explain the disparities in children’s dietary intake patterns according to skin colour in our sample. Recent studies showed that the availability of foods at the household level has an impact on children’s food consumption [38, 39]. Unfortunately, we were not able to measure household food availability and the number of meals per day in this cohort. Further research would be useful to help us to understand the mechanisms underlying the differences in dietary intake by skin colour in Brazil.

### Early feeding practices

Breastfeeding duration has been positively associated with healthy dietary patterns in children. A study conducted with 6 year-old children in the US found that breastfeeding duration was associated with higher intake of fruits and lower intake of sugar-sweetened beverages [40]. Similarly, another study conducted in Australia found that breastfeeding duration was associated with a healthy dietary pattern in 2–8 year-old children [41]. We found similar results, since children who were exclusively breastfed for less than 3 months and who started complementary feeding before 4 months of age consumed more *snacks and treats* (and more *coffee and bread*) and less *fruits and vegetables*, suggesting that early weaning along with early introduction of complementary feeding is related to ‘unhealthier’ feeding habits at 6 years. Some studies have raised an hypothesis where breastfeeding can improve children’s acceptability to new foods [42, 43], increasing the variety in children’s dietary intake.

### BMI z-score at 6 years

We expected that obese children would have higher consumption in the majority of dietary components, mainly in those indicative of unhealthier diet (such as *snacks and treats*), and lower consumption of *fruits and vegetables*. Nonetheless, we observed that overweight and obese children presented lower intake of four out of seven dietary components, including *snacks and treats*. As children’s consumption was collected based on mothers’ report, a possible explanation for this result is the fact that mothers of obese children, aware about their obesity status, could be underestimating their food consumption. Moreover, both food consumption and BMI status were collected at the same age and this association may be affected by reverse causality.

Extra analyses treating BMI as outcome showed that high intake of *coffee and bread* was negatively associated with BMI z-score at 6 years, while moderate and high consumption of *cheese and processed meats* was positively associated with BMI z-score at 6 years. This finding is interesting to note, as recent investigations in Brazil have shown that ultra-processed foods may play a key role in the obesity epidemic, providing around 30 % of daily energy intake in adolescents and adults [44, 45]. In addition, a recent study conducted in the 1982 Pelotas cohort study showed that high intake of processed foods was positively correlated with intake of sodium, cholesterol, and fats [46]. Therefore, our results could indicate that high intake of ultra-processed foods, characterized by ready-to-eat foods with minimal or no preparation [47], may also be important in the development of childhood obesity in this cohort. But again we must be cautious with interpretation due to reverse causality. More studies are needed in order to assess the longitudinal effect of food intake patterns on BMI and adiposity status.

### Strengths and limitations

Some strengths of this study are the cohort’s size and longevity, the low losses and refusal rates (only 9.8 %), and regular data collection that helped to minimize biases. Moreover, the FFQ used in our study was validated based on three 24-h dietary recalls. Food frequency questionnaires are very useful to investigate food consumption in large population-based studies as this method allows collection of complex dietary intake information in a simple, cheap and time-effective way [48].

Regarding PCA, the seven independent components identified in our study explained almost 50 % of the variation in children’s food consumption. The variance preserved in other studies which used PCA to analyse dietary intake patterns ranged from 26.8 up to 48.4 % [9, 11, 13, 19], which puts our results along with others with the highest percentage of variation explained. In addition, the seven components that we selected are visibly distinct by the type of food group that loaded on it (Table 2). PCA has been successfully used to describe the feeding practices of children in this cohort (at earlier ages) [19] and in other studies [9, 10, 15].

On the other hand, the 12 months recall period for the FFQ can be considered a limitation of our study, since it is a very long time frame and can result in recall bias. In addition, the FFQ was administered to the child’s mother, representing an indirect measure of the child’s food consumption, which may result in measurement error.

## Conclusions

In conclusion, our study added evidence that dietary intake patterns in children are strongly influenced by socioeconomic characteristics, since six out seven dietary components of children’s dietary intake patterns were associated with SEP. Furthermore, younger maternal age at birth as well as early weaning and early introduction of complementary feeding appear to be related with an ‘unhealthier’ dietary intake patterns, which may cause adverse outcomes in the long-term. Finally, overweight and obese children presented lower intake of four out of seven dietary components but as food consumption and BMI information were collected at the same time, further studies would be interesting to understand the longitudinal effect of children’s feeding practices on BMI and adiposity.

## Additional files

Additional file 1: Table S2. Multiple linear regression between dietary intake components and BMI z-score at 6 years (BMI as the outcome). Pelotas 2004 birth cohort study (*N* = 3,427). (DOCX 15 kb)

Additional file 2: Table S1. Fifty-four food items of the FFQ categorized according to nutritional characteristics (The 22 groups included in PCA).

Additional file 3: Figure S1. Direct acyclic graph of the effect of socioeconomic and demographic characteristics, early feeding practices and BMI z-score on dietary intake patterns (Demographic characteristics = maternal age at birth, and child’s sex and skin colour; Early feeding practices = exclusive breastfeeding duration and age of introduction of complementary foods).

## Abbreviations

ALSPAC: Avon longitudinal study of parents and children
BMI: Body mass index
CI: Confidence intervals
FFQ: Food frequency questionnaire
IEN: Brazilian National Economic Index
PCA: Principal component analysis
s.d.: Standard deviation
SEP: Socioeconomic position
WHO: World Health Organization
