# Supplementary material for: Dietary intake patterns of children aged 6 years and their association with socioeconomic and demographic characteristics, early feeding practices and body mass index
Source: BMC Public Health. 2016 Oct 6;16:1055. doi: 10.1186/s12889-016-3725-2 (PMC5052805; doi:10.1186/s12889-016-3725-2)
Supplement: Additional file 1: Table S2. — Multiple linear regression between dietary intake components and BMI z-score at 6 years (BMI as the outcome). Pelotas 2004 birth cohort study (N = 3,427). (DOCX 15 kb) [file 12889_2016_3725_MOESM1_ESM.docx]

**Supplementary table 2.** Multiple linear regression between dietary intake components and BMI z-score at 6 years (BMI as the outcome). Pelotas 2004 birth cohort study (N=3,427).

| **Consumption of dietary intake components** | **BMI z-score at 6 years** | **p-value** |
| --- | --- | --- |
|  | **β (CI95%)** |  |
| **Fruits and Vegetables** |  | ***0.169*** |
| Low (1^st^ tertile) | 0.00 |  |
| Intermediate (2^nd^ tertile) | -0.08 (-0.21; 0.05) |  |
| High (3^rd^ tertile) | -0.10 (-0.23; 0.04) |  |
|  |  |  |
| **Snack and treats** |  | ***0.161*** |
| Low (1^st^ tertile) | 0.00 |  |
| Intermediate (2^nd^ tertile) | -0.11 (-0.24; 0.02) |  |
| High (3^rd^ tertile) | -0.10 (-0.26; 0.05) |  |
|  |  |  |
| **Coffee and bread** |  | ***<0.001*** |
| Low (1^st^ tertile) | 0.00 |  |
| Intermediate (2^nd^ tertile) | -0.12 (-0.25; 0.02) |  |
| High (3^rd^ tertile) | -0.25 (-0.40; -0.11) |  |
|  |  |  |
| **Milk** |  | ***0.995*** |
| Low (1^st^ tertile) | 0.00 |  |
| Intermediate (2^nd^ tertile) | 0.17 (0.03; 0.30) |  |
| High (3^rd^ tertile) | 0.01 (-0.13; 0.14) |  |
|  |  |  |
| **Cheese and processed meats** |  | ***<0.001*** |
| Low (1^st^ tertile) | 0.00 |  |
| Intermediate (2^nd^ tertile) | 0.18 (0.05; 0.30) |  |
| High (3^rd^ tertile) | 0.25 (0.12; 0.38) |  |
|  |  |  |
| **Rice and beans** |  | ***0.271*** |
| Low (1^st^ tertile) | 0.00 |  |
| Intermediate (2^nd^ tertile) | -0.10 (-0.23; 0.03) |  |
| High (3^rd^ tertile) | -0.07 (-0.21; 0.06) |  |
|  |  |  |
| **Carbohydrates** |  | ***0.138*** |
| Low (1^st^ tertile) | 0.00 |  |
| Intermediate (2^nd^ tertile) | 0.02 (-0.10; 0.15) |  |
| High (3^rd^ tertile) | -0.10 (-0.23; 0.03) |  |
